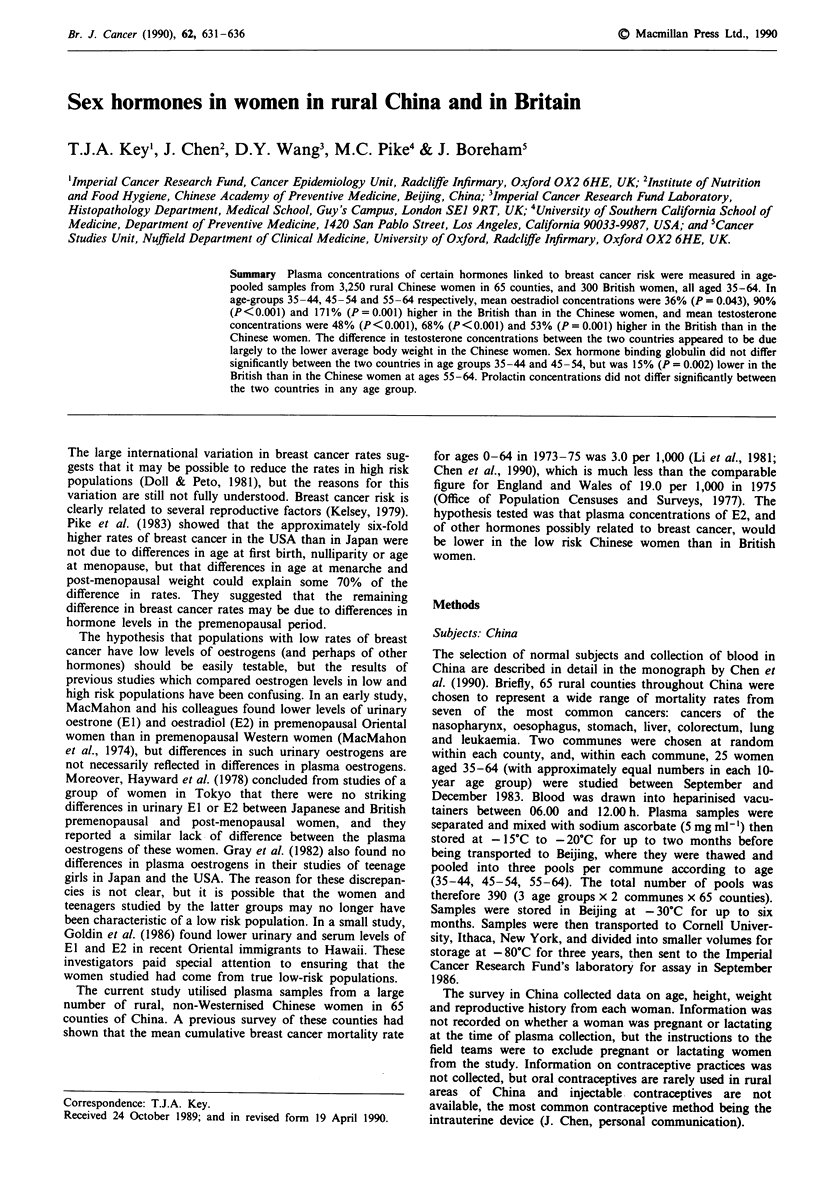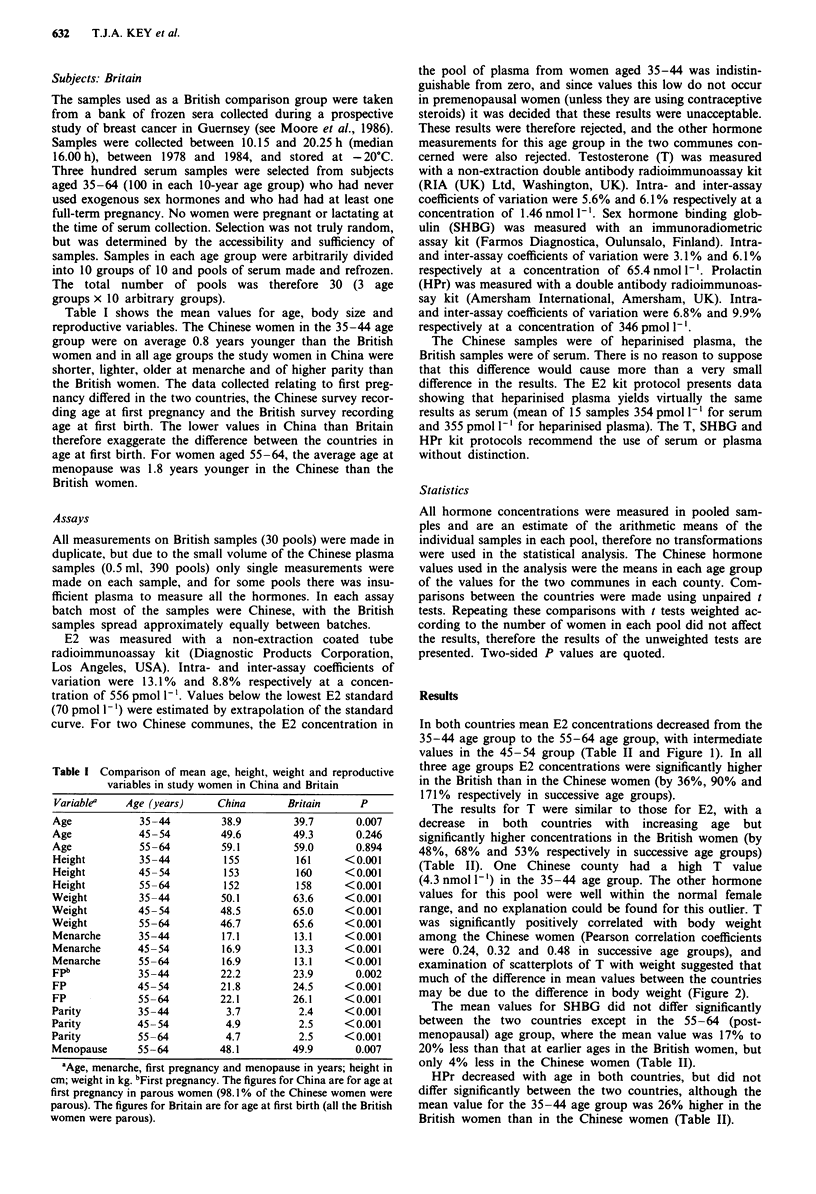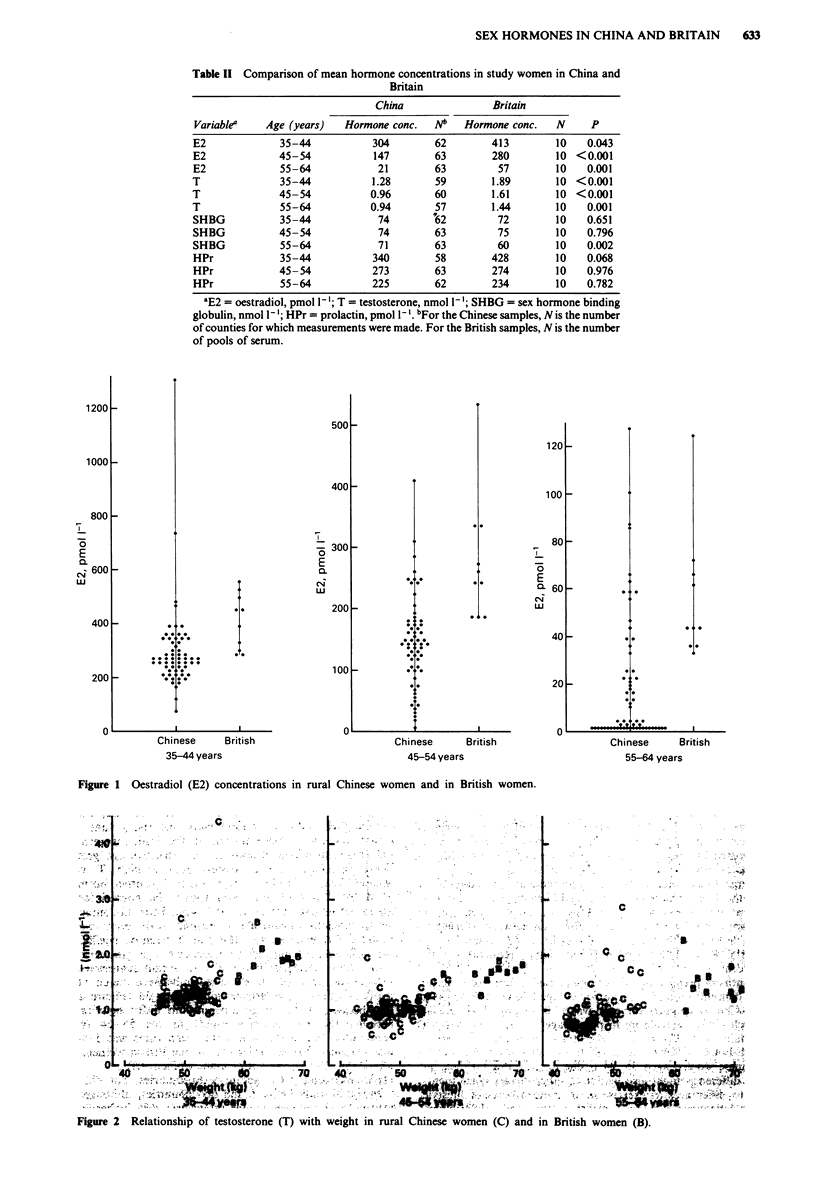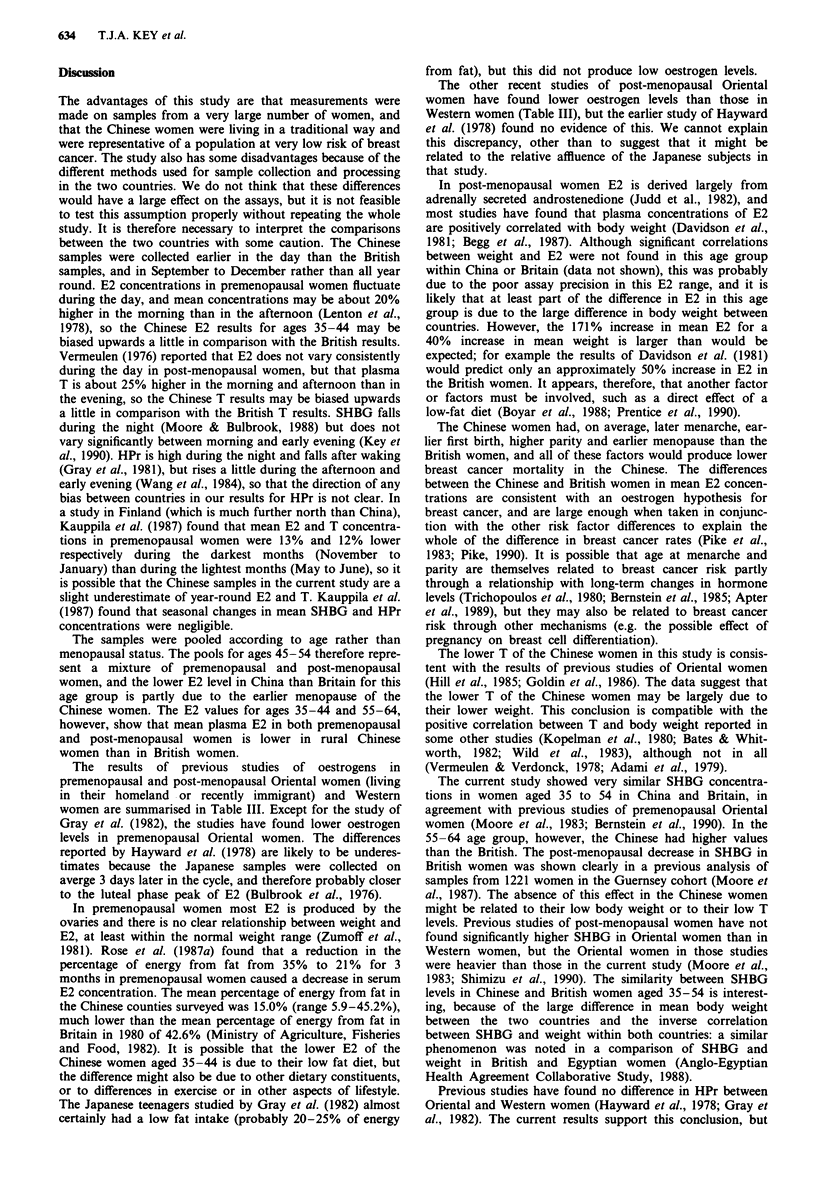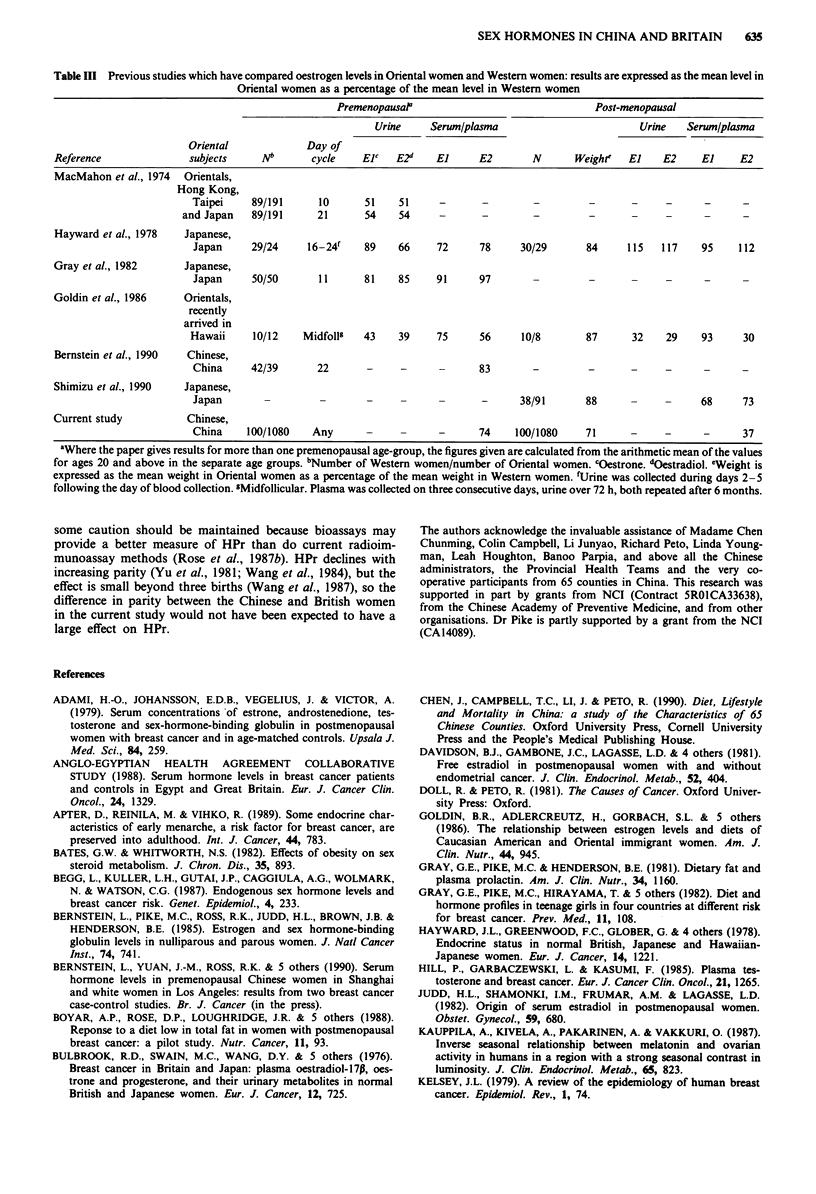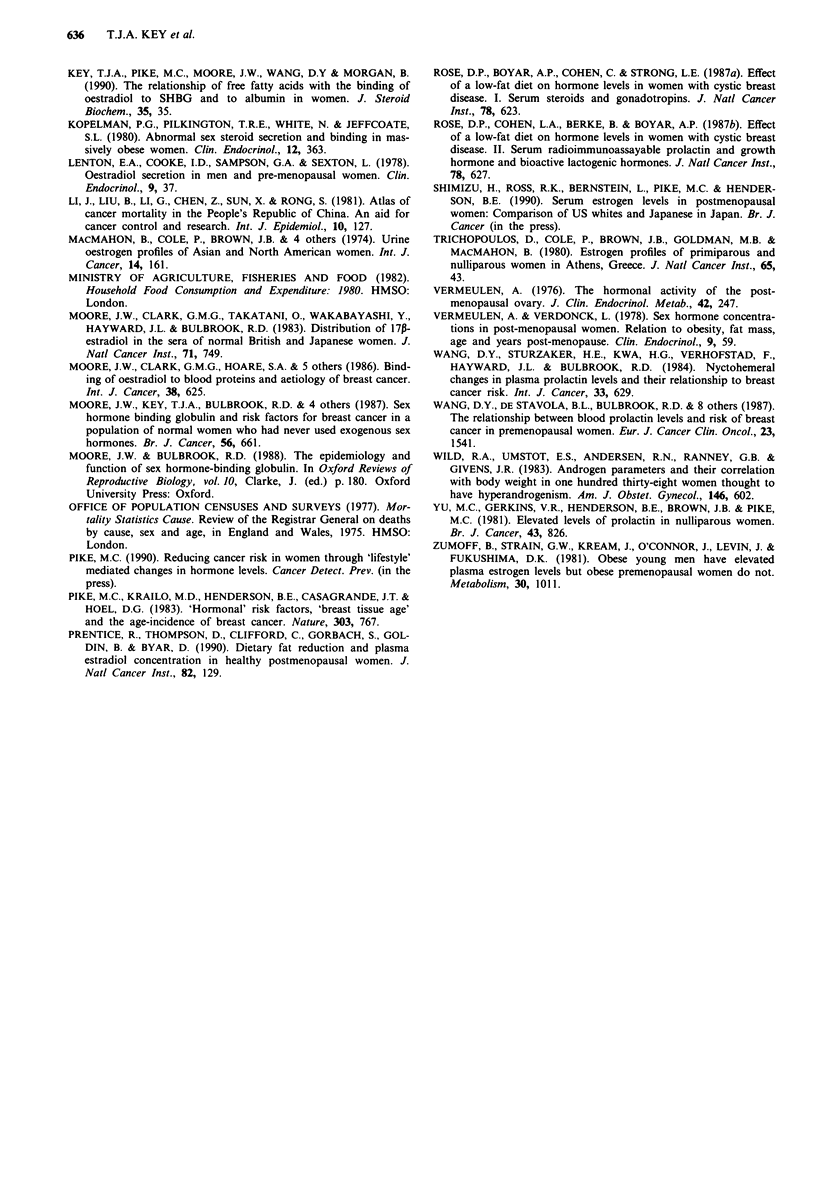# Sex hormones in women in rural China and in Britain.

**DOI:** 10.1038/bjc.1990.344

**Published:** 1990-10

**Authors:** T. J. Key, J. Chen, D. Y. Wang, M. C. Pike, J. Boreham

**Affiliations:** Imperial Cancer Research Fund, Cancer Epidemiology Unit, Radcliffe Infirmary, Oxford, UK.

## Abstract

Plasma concentrations of certain hormones linked to breast cancer risk were measured in age-pooled samples from 3,250 rural Chinese women in 65 counties, and 300 British women, all aged 35-64. In age-groups 35-44, 45-54 and 55-64 respectively, mean oestradiol concentrations were 36% (P = 0.043), 90% (P less than 0.001) and 171% (P = 0.001) higher in the British than in the Chinese women, and mean testosterone concentrations were 48% (P less than 0.001), 68% (P less than 0.001) and 53% (P = 0.001) higher in the British than in the Chinese women. The difference in testosterone concentrations between the two countries appeared to be due largely to the lower average body weight in the Chinese women. Sex hormone binding globulin did not differ significantly between the two countries in age groups 35-44 and 45-54, but was 15% (P = 0.002) lower in the British than in the Chinese women at ages 55-64. Prolactin concentrations did not differ significantly between the two countries in any age group.


Br. J. Cancer (1990), 62, 631-636 © Macmillan Press Ltd., 1990

### Sex hormones in women in rural China and in Britain

T.J.A. Key[1], J. Chen[2], D.Y. Wang[3], M.C. Pike[4] & J. Boreham[5]

[1]Imperial Cancer Research Fund, Cancer Epidemiology Unit, Radcliffe Infirmary, Oxford OX2 6HE, UK; [2]Institute of Nutrition and Food Hygiene, Chinese Academy of Preventive Medicine, Beijing, China; [3]Imperial Cancer Research Fund Laboratory, Histopathology Department, Medical School, Guy's Campus, London SE1 9RT, UK; [4]University of Southern California School of Medicine, Department of Preventive Medicine, 1420 San Pablo Street, Los Angeles, California 90033-9987, USA; and [5]Cancer Studies Unit, Nuffield Department of Clinical Medicine, University of Oxford, Radcliffe Infirmary, Oxford OX2 6HE, UK.

**Summary** Plasma concentrations of certain hormones linked to breast cancer risk were measured in age-pooled samples from 3,250 rural Chinese women in 65 counties, and 300 British women, all aged 35-64. In age-groups 35-44, 45-54 and 55-64 respectively, mean oestradiol concentrations were 36% ($P = 0.043$), 90% ($P<0.001$) and 171% ($P = 0.001$) higher in the British than in the Chinese women, and mean testosterone concentrations were 48% ($P<0.001$), 68% ($P<0.001$) and 53% ($P = 0.001$) higher in the British than in the Chinese women. The difference in testosterone concentrations between the two countries appeared to be due largely to the lower average body weight in the Chinese women. Sex hormone binding globulin did not differ significantly between the two countries in age groups 35-44 and 45-54, but was 15% ($P = 0.002$) lower in the British than in the Chinese women at ages 55-64. Prolactin concentrations did not differ significantly between the two countries in any age group.

The large international variation in breast cancer rates suggests that it may be possible to reduce the rates in high risk populations (Doll & Peto, 1981), but the reasons for this variation are still not fully understood. Breast cancer risk is clearly related to several reproductive factors (Kelsey, 1979). Pike *et al.* (1983) showed that the approximately six-fold higher rates of breast cancer in the USA than in Japan were not due to differences in age at first birth, nulliparity or age at menopause, but that differences in age at menarche and post-menopausal weight could explain some 70% of the difference in rates. They suggested that the remaining difference in breast cancer rates may be due to differences in hormone levels in the premenopausal period.

The hypothesis that populations with low rates of breast cancer have low levels of oestrogens (and perhaps of other hormones) should be easily testable, but the results of previous studies which compared oestrogen levels in low and high risk populations have been confusing. In an early study, MacMahon and his colleagues found lower levels of urinary oestrone (E1) and oestradiol (E2) in premenopausal Oriental women than in premenopausal Western women (MacMahon *et al.*, 1974), but differences in such urinary oestrogens are not necessarily reflected in differences in plasma oestrogens. Moreover, Hayward *et al.* (1978) concluded from studies of a group of women in Tokyo that there were no striking differences in urinary E1 or E2 between Japanese and British premenopausal and post-menopausal women, and they reported a similar lack of difference between the plasma oestrogens of these women. Gray *et al.* (1982) also found no differences in plasma oestrogens in their studies of teenage girls in Japan and the USA. The reason for these discrepancies is not clear, but it is possible that the women and teenagers studied by the latter groups may no longer have been characteristic of a low risk population. In a small study, Goldin *et al.* (1986) found lower urinary and serum levels of E1 and E2 in recent Oriental immigrants to Hawaii. These investigators paid special attention to ensuring that the women studied had come from true low-risk populations.

The current study utilised plasma samples from a large number of rural, non-Westernised Chinese women in 65 counties of China. A previous survey of these counties had shown that the mean cumulative breast cancer mortality rate for ages 0-64 in 1973-75 was 3.0 per 1,000 (Li *et al.*, 1981; Chen *et al.*, 1990), which is much less than the comparable figure for England and Wales of 19.0 per 1,000 in 1975 (Office of Population Censuses and Surveys, 1977). The hypothesis tested was that plasma concentrations of E2, and of other hormones possibly related to breast cancer, would be lower in the low risk Chinese women than in British women.

### Methods

### *Subjects: China*

The selection of normal subjects and collection of blood in China are described in detail in the monograph by Chen *et al.* (1990). Briefly, 65 rural counties throughout China were chosen to represent a wide range of mortality rates from seven of the most common cancers: cancers of the nasopharynx, oesophagus, stomach, liver, colorectum, lung and leukaemia. Two communes were chosen at random within each county, and, within each commune, 25 women aged 35-64 (with approximately equal numbers in each 10-year age group) were studied between September and December 1983. Blood was drawn into heparinised vacutainers between 06.00 and 12.00 h. Plasma samples were separated and mixed with sodium ascorbate (5 mg $ml^{-1}$) then stored at $-15°C$ to $-20°C$ for up to two months before being transported to Beijing, where they were thawed and pooled into three pools per commune according to age (35-44, 45-54, 55-64). The total number of pools was therefore 390 (3 age groups × 2 communes × 65 counties). Samples were stored in Beijing at $-30°C$ for up to six months. Samples were then transported to Cornell University, Ithaca, New York, and divided into smaller volumes for storage at $-80°C$ for three years, then sent to the Imperial Cancer Research Fund's laboratory for assay in September 1986.

The survey in China collected data on age, height, weight and reproductive history from each woman. Information was not recorded on whether a woman was pregnant or lactating at the time of plasma collection, but the instructions to the field teams were to exclude pregnant or lactating women from the study. Information on contraceptive practices was not collected, but oral contraceptives are rarely used in rural areas of China and injectable contraceptives are not available, the most common contraceptive method being the intrauterine device (J. Chen, personal communication).

Correspondence: T.J.A. Key.
Received 24 October 1989; and in revised form 19 April 1990.

*Subjects: Britain*

The samples used as a British comparison group were taken from a bank of frozen sera collected during a prospective study of breast cancer in Guernsey (see Moore *et al.*, 1986). Samples were collected between 10.15 and 20.25 h (median 16.00 h), between 1978 and 1984, and stored at −20°C. Three hundred serum samples were selected from subjects aged 35–64 (100 in each 10-year age group) who had never used exogenous sex hormones and who had had at least one full-term pregnancy. No women were pregnant or lactating at the time of serum collection. Selection was not truly random, but was determined by the accessibility and sufficiency of samples. Samples in each age group were arbitrarily divided into 10 groups of 10 and pools of serum made and refrozen. The total number of pools was therefore 30 (3 age groups × 10 arbitrary groups).

Table I shows the mean values for age, body size and reproductive variables. The Chinese women in the 35–44 age group were on average 0.8 years younger than the British women and in all age groups the study women in China were shorter, lighter, older at menarche and of higher parity than the British women. The data collected relating to first pregnancy differed in the two countries, the Chinese survey recording age at first pregnancy and the British survey recording age at first birth. The lower values in China than Britain therefore exaggerate the difference between the countries in age at first birth. For women aged 55–64, the average age at menopause was 1.8 years younger in the Chinese than the British women.

*Assays*

All measurements on British samples (30 pools) were made in duplicate, but due to the small volume of the Chinese plasma samples (0.5 ml, 390 pools) only single measurements were made on each sample, and for some pools there was insufficient plasma to measure all the hormones. In each assay batch most of the samples were Chinese, with the British samples spread approximately equally between batches.

E2 was measured with a non-extraction coated tube radioimmunoassay kit (Diagnostic Products Corporation, Los Angeles, USA). Intra- and inter-assay coefficients of variation were 13.1% and 8.8% respectively at a concentration of 556 pmol $l^{-1}$. Values below the lowest E2 standard (70 pmol $l^{-1}$) were estimated by extrapolation of the standard curve. For two Chinese communes, the E2 concentration in the pool of plasma from women aged 35–44 was indistinguishable from zero, and since values this low do not occur in premenopausal women (unless they are using contraceptive steroids) it was decided that these results were unacceptable. These results were therefore rejected, and the other hormone measurements for this age group in the two communes concerned were also rejected. Testosterone (T) was measured with a non-extraction double antibody radioimmunoassay kit (RIA (UK) Ltd, Washington, UK). Intra- and inter-assay coefficients of variation were 5.6% and 6.1% respectively at a concentration of 1.46 nmol $l^{-1}$. Sex hormone binding globulin (SHBG) was measured with an immunoradiometric assay kit (Farmos Diagnostica, Oulunsalo, Finland). Intra- and inter-assay coefficients of variation were 3.1% and 6.1% respectively at a concentration of 65.4 nmol $l^{-1}$. Prolactin (HPr) was measured with a double antibody radioimmunoassay kit (Amersham International, Amersham, UK). Intra- and inter-assay coefficients of variation were 6.8% and 9.9% respectively at a concentration of 346 pmol $l^{-1}$.

The Chinese samples were of heparinised plasma, the British samples were of serum. There is no reason to suppose that this difference would cause more than a very small difference in the results. The E2 kit protocol presents data showing that heparinised plasma yields virtually the same results as serum (mean of 15 samples 354 pmol $l^{-1}$ for serum and 355 pmol $l^{-1}$ for heparinised plasma). The T, SHBG and HPr kit protocols recommend the use of serum or plasma without distinction.

*Statistics*

All hormone concentrations were measured in pooled samples and are an estimate of the arithmetic means of the individual samples in each pool, therefore no transformations were used in the statistical analysis. The Chinese hormone values used in the analysis were the means in each age group of the values for the two communes in each county. Comparisons between the countries were made using unpaired *t* tests. Repeating these comparisons with *t* tests weighted according to the number of women in each pool did not affect the results, therefore the results of the unweighted tests are presented. Two-sided *P* values are quoted.

**Table I** Comparison of mean age, height, weight and reproductive variables in study women in China and Britain

| *Variable*[a] | *Age (years)* | *China* | *Britain* | *P* |
|---|---|---|---|---|
| Age | 35–44 | 38.9 | 39.7 | 0.007 |
| Age | 45–54 | 49.6 | 49.3 | 0.246 |
| Age | 55–64 | 59.1 | 59.0 | 0.894 |
| Height | 35–44 | 155 | 161 | <0.001 |
| Height | 45–54 | 153 | 160 | <0.001 |
| Height | 55–64 | 152 | 158 | <0.001 |
| Weight | 35–44 | 50.1 | 63.6 | <0.001 |
| Weight | 45–54 | 48.5 | 65.0 | <0.001 |
| Weight | 55–64 | 46.7 | 65.6 | <0.001 |
| Menarche | 35–44 | 17.1 | 13.1 | <0.001 |
| Menarche | 45–54 | 16.9 | 13.3 | <0.001 |
| Menarche | 55–64 | 16.9 | 13.1 | <0.001 |
| FP[b] | 35–44 | 22.2 | 23.9 | 0.002 |
| FP | 45–54 | 21.8 | 24.5 | <0.001 |
| FP | 55–64 | 22.1 | 26.1 | <0.001 |
| Parity | 35–44 | 3.7 | 2.4 | <0.001 |
| Parity | 45–54 | 4.9 | 2.5 | <0.001 |
| Parity | 55–64 | 4.7 | 2.5 | <0.001 |
| Menopause | 55–64 | 48.1 | 49.9 | 0.007 |

[a]Age, menarche, first pregnancy and menopause in years; height in cm; weight in kg. [b]First pregnancy. The figures for China are for age at first pregnancy in parous women (98.1% of the Chinese women were parous). The figures for Britain are for age at first birth (all the British women were parous).

### Results

In both countries mean E2 concentrations decreased from the 35–44 age group to the 55–64 age group, with intermediate values in the 45–54 group (Table II and Figure 1). In all three age groups E2 concentrations were significantly higher in the British than in the Chinese women (by 36%, 90% and 171% respectively in successive age groups).

The results for T were similar to those for E2, with a decrease in both countries with increasing age but significantly higher concentrations in the British women (by 48%, 68% and 53% respectively in successive age groups) (Table II). One Chinese county had a high T value (4.3 nmol $l^{-1}$) in the 35–44 age group. The other hormone values for this pool were well within the normal female range, and no explanation could be found for this outlier. T was significantly positively correlated with body weight among the Chinese women (Pearson correlation coefficients were 0.24, 0.32 and 0.48 in successive age groups), and examination of scatterplots of T with weight suggested that much of the difference in mean values between the countries may be due to the difference in body weight (Figure 2).

The mean values for SHBG did not differ significantly between the two countries except in the 55–64 (postmenopausal) age group, where the mean value was 17% to 20% less than that at earlier ages in the British women, but only 4% less in the Chinese women (Table II).

HPr decreased with age in both countries, but did not differ significantly between the two countries, although the mean value for the 35–44 age group was 26% higher in the British women than in the Chinese women (Table II).

**Table II** Comparison of mean hormone concentrations in study women in China and Britain

| | | China | | Britain | | |
|---|---|---|---|---|---|---|
| *Variable*[a] | *Age (years)* | *Hormone conc.* | *N*[b] | *Hormone conc.* | *N* | *P* |
| E2 | 35–44 | 304 | 62 | 413 | 10 | 0.043 |
| E2 | 45–54 | 147 | 63 | 280 | 10 | <0.001 |
| E2 | 55–64 | 21 | 63 | 57 | 10 | 0.001 |
| T | 35–44 | 1.28 | 59 | 1.89 | 10 | <0.001 |
| T | 45–54 | 0.96 | 60 | 1.61 | 10 | <0.001 |
| T | 55–64 | 0.94 | 57 | 1.44 | 10 | 0.001 |
| SHBG | 35–44 | 74 | 62 | 72 | 10 | 0.651 |
| SHBG | 45–54 | 74 | 63 | 75 | 10 | 0.796 |
| SHBG | 55–64 | 71 | 63 | 60 | 10 | 0.002 |
| HPr | 35–44 | 340 | 58 | 428 | 10 | 0.068 |
| HPr | 45–54 | 273 | 63 | 274 | 10 | 0.976 |
| HPr | 55–64 | 225 | 62 | 234 | 10 | 0.782 |

[a]E2 = oestradiol, pmol $l^{-1}$; T = testosterone, nmol $l^{-1}$; SHBG = sex hormone binding globulin, nmol $l^{-1}$; HPr = prolactin, pmol $l^{-1}$. [b]For the Chinese samples, *N* is the number of counties for which measurements were made. For the British samples, *N* is the number of pools of serum.

**Figure 1** Oestradiol (E2) concentrations in rural Chinese women and in British women.

**Figure 2** Relationship of testosterone (T) with weight in rural Chinese women (C) and in British women (B).

### Discussion

The advantages of this study are that measurements were made on samples from a very large number of women, and that the Chinese women were living in a traditional way and were representative of a population at very low risk of breast cancer. The study also has some disadvantages because of the different methods used for sample collection and processing in the two countries. We do not think that these differences would have a large effect on the assays, but it is not feasible to test this assumption properly without repeating the whole study. It is therefore necessary to interpret the comparisons between the two countries with some caution. The Chinese samples were collected earlier in the day than the British samples, and in September to December rather than all year round. E2 concentrations in premenopausal women fluctuate during the day, and mean concentrations may be about 20% higher in the morning than in the afternoon (Lenton *et al.*, 1978), so the Chinese E2 results for ages 35–44 may be biased upwards a little in comparison with the British results. Vermeulen (1976) reported that E2 does not vary consistently during the day in post-menopausal women, but that plasma T is about 25% higher in the morning and afternoon than in the evening, so the Chinese T results may be biased upwards a little in comparison with the British T results. SHBG falls during the night (Moore & Bulbrook, 1988) but does not vary significantly between morning and early evening (Key *et al.*, 1990). HPr is high during the night and falls after waking (Gray *et al.*, 1981), but rises a little during the afternoon and early evening (Wang *et al.*, 1984), so that the direction of any bias between countries in our results for HPr is not clear. In a study in Finland (which is much further north than China), Kauppila *et al.* (1987) found that mean E2 and T concentrations in premenopausal women were 13% and 12% lower respectively during the darkest months (November to January) than during the lightest months (May to June), so it is possible that the Chinese samples in the current study are a slight underestimate of year-round E2 and T. Kauppila *et al.* (1987) found that seasonal changes in mean SHBG and HPr concentrations were negligible.

The samples were pooled according to age rather than menopausal status. The pools for ages 45–54 therefore represent a mixture of premenopausal and post-menopausal women, and the lower E2 level in China than Britain for this age group is partly due to the earlier menopause of the Chinese women. The E2 values for ages 35–44 and 55–64, however, show that mean plasma E2 in both premenopausal and post-menopausal women is lower in rural Chinese women than in British women.

The results of previous studies of oestrogens in premenopausal and post-menopausal Oriental women (living in their homeland or recently immigrant) and Western women are summarised in Table III. Except for the study of Gray *et al.* (1982), the studies have found lower oestrogen levels in premenopausal Oriental women. The differences reported by Hayward *et al.* (1978) are likely to be underestimates because the Japanese samples were collected on averge 3 days later in the cycle, and therefore probably closer to the luteal phase peak of E2 (Bulbrook *et al.*, 1976).

In premenopausal women most E2 is produced by the ovaries and there is no clear relationship between weight and E2, at least within the normal weight range (Zumoff *et al.*, 1981). Rose *et al.* (1987*a*) found that a reduction in the percentage of energy from fat from 35% to 21% for 3 months in premenopausal women caused a decrease in serum E2 concentration. The mean percentage of energy from fat in the Chinese counties surveyed was 15.0% (range 5.9–45.2%), much lower than the mean percentage of energy from fat in Britain in 1980 of 42.6% (Ministry of Agriculture, Fisheries and Food, 1982). It is possible that the lower E2 of the Chinese women aged 35–44 is due to their low fat diet, but the difference might also be due to other dietary constituents, or to differences in exercise or in other aspects of lifestyle. The Japanese teenagers studied by Gray *et al.* (1982) almost certainly had a low fat intake (probably 20–25% of energy from fat), but this did not produce low oestrogen levels.

The other recent studies of post-menopausal Oriental women have found lower oestrogen levels than those in Western women (Table III), but the earlier study of Hayward *et al.* (1978) found no evidence of this. We cannot explain this discrepancy, other than to suggest that it might be related to the relative affluence of the Japanese subjects in that study.

In post-menopausal women E2 is derived largely from adrenally secreted androstenedione (Judd et al., 1982), and most studies have found that plasma concentrations of E2 are positively correlated with body weight (Davidson *et al.*, 1981; Begg *et al.*, 1987). Although significant correlations between weight and E2 were not found in this age group within China or Britain (data not shown), this was probably due to the poor assay precision in this E2 range, and it is likely that at least part of the difference in E2 in this age group is due to the large difference in body weight between countries. However, the 171% increase in mean E2 for a 40% increase in mean weight is larger than would be expected; for example the results of Davidson *et al.* (1981) would predict only an approximately 50% increase in E2 in the British women. It appears, therefore, that another factor or factors must be involved, such as a direct effect of a low-fat diet (Boyar *et al.*, 1988; Prentice *et al.*, 1990).

The Chinese women had, on average, later menarche, earlier first birth, higher parity and earlier menopause than the British women, and all of these factors would produce lower breast cancer mortality in the Chinese. The differences between the Chinese and British women in mean E2 concentrations are consistent with an oestrogen hypothesis for breast cancer, and are large enough when taken in conjunction with the other risk factor differences to explain the whole of the difference in breast cancer rates (Pike *et al.*, 1983; Pike, 1990). It is possible that age at menarche and parity are themselves related to breast cancer risk partly through a relationship with long-term changes in hormone levels (Trichopoulos *et al.*, 1980; Bernstein *et al.*, 1985; Apter *et al.*, 1989), but they may also be related to breast cancer risk through other mechanisms (e.g. the possible effect of pregnancy on breast cell differentiation).

The lower T of the Chinese women in this study is consistent with the results of previous studies of Oriental women (Hill *et al.*, 1985; Goldin *et al.*, 1986). The data suggest that the lower T of the Chinese women may be largely due to their lower weight. This conclusion is compatible with the positive correlation between T and body weight reported in some other studies (Kopelman *et al.*, 1980; Bates & Whitworth, 1982; Wild *et al.*, 1983), although not in all (Vermeulen & Verdonck, 1978; Adami *et al.*, 1979).

The current study showed very similar SHBG concentrations in women aged 35 to 54 in China and Britain, in agreement with previous studies of premenopausal Oriental women (Moore *et al.*, 1983; Bernstein *et al.*, 1990). In the 55–64 age group, however, the Chinese had higher values than the British. The post-menopausal decrease in SHBG in British women was shown clearly in a previous analysis of samples from 1221 women in the Guernsey cohort (Moore *et al.*, 1987). The absence of this effect in the Chinese women might be related to their low body weight or to their low T levels. Previous studies of post-menopausal women have not found significantly higher SHBG in Oriental women than in Western women, but the Oriental women in those studies were heavier than those in the current study (Moore *et al.*, 1983; Shimizu *et al.*, 1990). The similarity between SHBG levels in Chinese and British women aged 35–54 is interesting, because of the large difference in mean body weight between the two countries and the inverse correlation between SHBG and weight within both countries: a similar phenomenon was noted in a comparison of SHBG and weight in British and Egyptian women (Anglo-Egyptian Health Agreement Collaborative Study, 1988).

Previous studies have found no difference in HPr between Oriental and Western women (Hayward *et al.*, 1978; Gray *et al.*, 1982). The current results support this conclusion, but

**Table III** Previous studies which have compared oestrogen levels in Oriental women and Western women: results are expressed as the mean level in Oriental women as a percentage of the mean level in Western women

| | | *Premenopausal*[a] | | | | | | *Post-menopausal* | | | | | |
|---|---|---|---|---|---|---|---|---|---|---|---|---|---|
| | | | | *Urine* | | *Serum/plasma* | | | | *Urine* | | *Serum/plasma* | |
| *Reference* | *Oriental subjects* | *N*[b] | *Day of cycle* | *E1*[c] | *E2*[d] | *E1* | *E2* | *N* | *Weight*[e] | *E1* | *E2* | *E1* | *E2* |
| MacMahon *et al.*, 1974 | Orientals, Hong Kong, Taipei | 89/191 | 10 | 51 | 51 | – | – | – | – | – | – | – | – |
| | and Japan | 89/191 | 21 | 54 | 54 | – | – | – | – | – | – | – | – |
| Hayward *et al.*, 1978 | Japanese, Japan | 29/24 | 16–24[f] | 89 | 66 | 72 | 78 | 30/29 | 84 | 115 | 117 | 95 | 112 |
| Gray *et al.*, 1982 | Japanese, Japan | 50/50 | 11 | 81 | 85 | 91 | 97 | – | – | – | – | – | – |
| Goldin *et al.*, 1986 | Orientals, recently arrived in Hawaii | 10/12 | Midfoll[g] | 43 | 39 | 75 | 56 | 10/8 | 87 | 32 | 29 | 93 | 30 |
| Bernstein *et al.*, 1990 | Chinese, China | 42/39 | 22 | – | – | – | 83 | – | – | – | – | – | – |
| Shimizu *et al.*, 1990 | Japanese, Japan | – | – | – | – | – | – | 38/91 | 88 | – | – | 68 | 73 |
| Current study | Chinese, China | 100/1080 | Any | – | – | – | 74 | 100/1080 | 71 | – | – | – | 37 |

[a]Where the paper gives results for more than one premenopausal age-group, the figures given are calculated from the arithmetic mean of the values for ages 20 and above in the separate age groups. [b]Number of Western women/number of Oriental women. [c]Oestrone. [d]Oestradiol. [e]Weight is expressed as the mean weight in Oriental women as a percentage of the mean weight in Western women. [f]Urine was collected during days 2–5 following the day of blood collection. [g]Midfollicular. Plasma was collected on three consecutive days, urine over 72 h, both repeated after 6 months.

some caution should be maintained because bioassays may provide a better measure of HPr than do current radioimmunoassay methods (Rose *et al.*, 1987*b*). HPr declines with increasing parity (Yu *et al.*, 1981; Wang *et al.*, 1984), but the effect is small beyond three births (Wang *et al.*, 1987), so the difference in parity between the Chinese and British women in the current study would not have been expected to have a large effect on HPr.

The authors acknowledge the invaluable assistance of Madame Chen Chunming, Colin Campbell, Li Junyao, Richard Peto, Linda Youngman, Leah Houghton, Banoo Parpia, and above all the Chinese administrators, the Provincial Health Teams and the very cooperative participants from 65 counties in China. This research was supported in part by grants from NCI (Contract 5R01CA33638), from the Chinese Academy of Preventive Medicine, and from other organisations. Dr Pike is partly supported by a grant from the NCI (CA14089).

### References

ADAMI, H.-O., JOHANSSON, E.D.B., VEGELIUS, J. & VICTOR, A. (1979). Serum concentrations of estrone, androstenedione, testosterone and sex-hormone-binding globulin in postmenopausal women with breast cancer and in age-matched controls. *Upsala J. Med. Sci.*, **84**, 259.

ANGLO-EGYPTIAN HEALTH AGREEMENT COLLABORATIVE STUDY (1988). Serum hormone levels in breast cancer patients and controls in Egypt and Great Britain. *Eur. J. Cancer Clin. Oncol.*, **24**, 1329.

APTER, D., REINILA, M. & VIHKO, R. (1989). Some endocrine characteristics of early menarche, a risk factor for breast cancer, are preserved into adulthood. *Int. J. Cancer*, **44**, 783.

BATES, G.W. & WHITWORTH, N.S. (1982). Effects of obesity on sex steroid metabolism. *J. Chron. Dis.*, **35**, 893.

BEGG, L., KULLER, L.H., GUTAI, J.P., CAGGIULA, A.G., WOLMARK, N. & WATSON, C.G. (1987). Endogenous sex hormone levels and breast cancer risk. *Genet. Epidemiol.*, **4**, 233.

BERNSTEIN, L., PIKE, M.C., ROSS, R.K., JUDD, H.L., BROWN, J.B. & HENDERSON, B.E. (1985). Estrogen and sex hormone-binding globulin levels in nulliparous and parous women. *J. Natl Cancer Inst.*, **74**, 741.

BERNSTEIN, L., YUAN, J.-M., ROSS, R.K. & 5 others (1990). Serum hormone levels in premenopausal Chinese women in Shanghai and white women in Los Angeles: results from two breast cancer case-control studies. *Br. J. Cancer* (in the press).

BOYAR, A.P., ROSE, D.P., LOUGHRIDGE, J.R. & 5 others (1988). Reponse to a diet low in total fat in women with postmenopausal breast cancer: a pilot study. *Nutr. Cancer*, **11**, 93.

BULBROOK, R.D., SWAIN, M.C., WANG, D.Y. & 5 others (1976). Breast cancer in Britain and Japan: plasma oestradiol-17β, oestrone and progesterone, and their urinary metabolites in normal British and Japanese women. *Eur. J. Cancer*, **12**, 725.

CHEN, J., CAMPBELL, T.C., LI, J. & PETO, R. (1990). *Diet, Lifestyle and Mortality in China: a study of the Characteristics of 65 Chinese Counties.* Oxford University Press, Cornell University Press and the People's Medical Publishing House.

DAVIDSON, B.J., GAMBONE, J.C., LAGASSE, L.D. & 4 others (1981). Free estradiol in postmenopausal women with and without endometrial cancer. *J. Clin. Endocrinol. Metab.*, **52**, 404.

DOLL, R. & PETO, R. (1981). *The Causes of Cancer*. Oxford University Press: Oxford.

GOLDIN, B.R., ADLERCREUTZ, H., GORBACH, S.L. & 5 others (1986). The relationship between estrogen levels and diets of Caucasian American and Oriental immigrant women. *Am. J. Clin. Nutr.*, **44**, 945.

GRAY, G.E., PIKE, M.C. & HENDERSON, B.E. (1981). Dietary fat and plasma prolactin. *Am. J. Clin. Nutr.*, **34**, 1160.

GRAY, G.E., PIKE, M.C., HIRAYAMA, T. & 5 others (1982). Diet and hormone profiles in teenage girls in four countries at different risk for breast cancer. *Prev. Med.*, **11**, 108.

HAYWARD, J.L., GREENWOOD, F.C., GLOBER, G. & 4 others (1978). Endocrine status in normal British, Japanese and Hawaiian-Japanese women. *Eur. J. Cancer*, **14**, 1221.

HILL, P., GARBACZEWSKI, L. & KASUMI, F. (1985). Plasma testosterone and breast cancer. *Eur. J. Cancer Clin. Oncol.*, **21**, 1265.

JUDD, H.L., SHAMONKI, I.M., FRUMAR, A.M. & LAGASSE, L.D. (1982). Origin of serum estradiol in postmenopausal women. *Obstet. Gynecol.*, **59**, 680.

KAUPPILA, A., KIVELA, A., PAKARINEN, A. & VAKKURI, O. (1987). Inverse seasonal relationship between melatonin and ovarian activity in humans in a region with a strong seasonal contrast in luminosity. *J. Clin. Endocrinol. Metab.*, **65**, 823.

KELSEY, J.L. (1979). A review of the epidemiology of human breast cancer. *Epidemiol. Rev.*, **1**, 74.

KEY, T.J.A., PIKE, M.C., MOORE, J.W., WANG, D.Y & MORGAN, B. (1990). The relationship of free fatty acids with the binding of oestradiol to SHBG and to albumin in women. *J. Steroid Biochem.*, **35**, 35.

KOPELMAN, P.G., PILKINGTON, T.R.E., WHITE, N. & JEFFCOATE, S.L. (1980). Abnormal sex steroid secretion and binding in massively obese women. *Clin. Endocrinol.*, **12**, 363.

LENTON, E.A., COOKE, I.D., SAMPSON, G.A. & SEXTON, L. (1978). Oestradiol secretion in men and pre-menopausal women. *Clin. Endocrinol.*, **9**, 37.

LI, J., LIU, B., LI, G., CHEN, Z., SUN, X. & RONG, S. (1981). Atlas of cancer mortality in the People's Republic of China. An aid for cancer control and research. *Int. J. Epidemiol.*, **10**, 127.

MACMAHON, B., COLE, P., BROWN, J.B. & 4 others (1974). Urine oestrogen profiles of Asian and North American women. *Int. J. Cancer*, **14**, 161.

MINISTRY OF AGRICULTURE, FISHERIES AND FOOD (1982). *Household Food Consumption and Expenditure: 1980*. HMSO: London.

MOORE, J.W., CLARK, G.M.G., TAKATANI, O., WAKABAYASHI, Y., HAYWARD, J.L. & BULBROOK, R.D. (1983). Distribution of 17β-estradiol in the sera of normal British and Japanese women. *J. Natl Cancer Inst.*, **71**, 749.

MOORE, J.W., CLARK, G.M.G., HOARE, S.A. & 5 others (1986). Binding of oestradiol to blood proteins and aetiology of breast cancer. *Int. J. Cancer*, **38**, 625.

MOORE, J.W., KEY, T.J.A., BULBROOK, R.D. & 4 others (1987). Sex hormone binding globulin and risk factors for breast cancer in a population of normal women who had never used exogenous sex hormones. *Br. J. Cancer*, **56**, 661.

MOORE, J.W. & BULBROOK, R.D. (1988). The epidemiology and function of sex hormone-binding globulin. In *Oxford Reviews of Reproductive Biology, vol. 10*, Clarke, J. (ed.) p. 180. Oxford University Press: Oxford.

OFFICE OF POPULATION CENSUSES AND SURVEYS (1977). *Mortality Statistics Cause*. Review of the Registrar General on deaths by cause, sex and age, in England and Wales, 1975. HMSO: London.

PIKE, M.C. (1990). Reducing cancer risk in women through 'lifestyle' mediated changes in hormone levels. *Cancer Detect. Prev.* (in the press).

PIKE, M.C., KRAILO, M.D., HENDERSON, B.E., CASAGRANDE, J.T. & HOEL, D.G. (1983). 'Hormonal' risk factors, 'breast tissue age' and the age-incidence of breast cancer. *Nature*, **303**, 767.

PRENTICE, R., THOMPSON, D., CLIFFORD, C., GORBACH, S., GOLDIN, B. & BYAR, D. (1990). Dietary fat reduction and plasma estradiol concentration in healthy postmenopausal women. *J. Natl Cancer Inst.*, **82**, 129.

ROSE, D.P., BOYAR, A.P., COHEN, C. & STRONG, L.E. (1987*a*). Effect of a low-fat diet on hormone levels in women with cystic breast disease. I. Serum steroids and gonadotropins. *J. Natl Cancer Inst.*, **78**, 623.

ROSE, D.P., COHEN, L.A., BERKE, B. & BOYAR, A.P. (1987*b*). Effect of a low-fat diet on hormone levels in women with cystic breast disease. II. Serum radioimmunoassayable prolactin and growth hormone and bioactive lactogenic hormones. *J. Natl Cancer Inst.*, **78**, 627.

SHIMIZU, H., ROSS, R.K., BERNSTEIN, L., PIKE, M.C. & HENDERSON, B.E. (1990). Serum estrogen levels in postmenopausal women: Comparison of US whites and Japanese in Japan. *Br. J. Cancer* (in the press).

TRICHOPOULOS, D., COLE, P., BROWN, J.B., GOLDMAN, M.B. & MACMAHON, B. (1980). Estrogen profiles of primiparous and nulliparous women in Athens, Greece. *J. Natl Cancer Inst.*, **65**, 43.

VERMEULEN, A. (1976). The hormonal activity of the postmenopausal ovary. *J. Clin. Endocrinol. Metab.*, **42**, 247.

VERMEULEN, A. & VERDONCK, L. (1978). Sex hormone concentrations in post-menopausal women. Relation to obesity, fat mass, age and years post-menopause. *Clin. Endocrinol.*, **9**, 59.

WANG, D.Y., STURZAKER, H.E., KWA, H.G., VERHOFSTAD, F., HAYWARD, J.L. & BULBROOK, R.D. (1984). Nyctohemeral changes in plasma prolactin levels and their relationship to breast cancer risk. *Int. J. Cancer*, **33**, 629.

WANG, D.Y., DE STAVOLA, B.L., BULBROOK, R.D. & 8 others (1987). The relationship between blood prolactin levels and risk of breast cancer in premenopausal women. *Eur. J. Cancer Clin. Oncol.*, **23**, 1541.

WILD, R.A., UMSTOT, E.S., ANDERSEN, R.N., RANNEY, G.B. & GIVENS, J.R. (1983). Androgen parameters and their correlation with body weight in one hundred thirty-eight women thought to have hyperandrogenism. *Am. J. Obstet. Gynecol.*, **146**, 602.

YU, M.C., GERKINS, V.R., HENDERSON, B.E., BROWN, J.B. & PIKE, M.C. (1981). Elevated levels of prolactin in nulliparous women. *Br. J. Cancer*, **43**, 826.

ZUMOFF, B., STRAIN, G.W., KREAM, J., O'CONNOR, J., LEVIN, J. & FUKUSHIMA, D.K. (1981). Obese young men have elevated plasma estrogen levels but obese premenopausal women do not. *Metabolism*, **30**, 1011.